# Immune-related response evaluations during immune-checkpoint inhibitor therapy: establishing a “common language” for the new arena of cancer treatment

**DOI:** 10.1186/s40425-016-0134-0

**Published:** 2016-06-21

**Authors:** Mizuki Nishino

**Affiliations:** Department of Radiology, Brigham and Women’s Hospital and Dana-Farber Cancer Institute, 450 Brookline Ave., Boston, MA 02215 USA

## Abstract

The recent study by Hodi et al. published in the Journal of Clinical Oncology has evaluated unconventional response patterns during PD-1 inhibitor therapy using immune-related response criteria (irRC) in comparison with RECIST1.1, which constitutes an important step to further understand immune-related response phenomena. This commentary discusses the key observations in the study in terms of their implications and pitfalls, and describes unmet needs that remain to be addressed. The article also emphasizes the important role of tumor response criteria as a “common language” to describe the results of cancer treatment, and discusses future directions for further advances of the field of immuno-oncology.

Unconventional tumor response patterns associated with immune-checkpoint blockade provide challenges for evaluations of treatment benefits in patients treated with immune-checkpoint inhibitors. In the recent JCO article by Hodi et al., atypical response patterns were evaluated using immune-related response criteria (irRC) by a retrospective analysis of 327 melanoma patients treated with PD-1 inhibitor pembrolizumab therapy [1]. This is the first study that evaluated immune-related responses in a large number of patients treated with PD-1 inhibitor therapy and reported the overall survival data in correlation with irRC and RECIST assessments. Their results indicated that the conventional RECIST assessment alone may underestimate the benefit of PD-1 inhibitor therapy in a subset of patients, supporting a need of immune-related response evaluation strategy that has been increasingly recognized among investigators in immuno-oncology community. The study has provided important and insightful observations and presented remaining and emerging challenges. Further discussions of some of the key observations help to understand the implications and pitfalls, and to develop strategies that address the challenges. Of note, tumor response criteria should serve as a “common language” to describe treatment results and provide a basis for advances in cancer therapy [2–4], and therefore the detailed methods of evaluating and defining immune-related responses deserve careful review to discuss the implications of the study on further growth of immuno-oncology community.

Response patterns unique to immune-checkpoint inhibitor therapy can be noted i) after an initial increase of tumor burden or ii) during or after the appearance of new lesions [5–7]. The phenomena are termed “pseudoprogression”, because they would be classified as progressive disease (PD) by conventional RECIST [5–9]. To capture these unconventional response patterns, irRC was proposed in 2009 with the key features including 1) requirement of confirmation of PD on two consecutive scans at least 4 weeks apart, and 2) inclusion of new lesion measurements to the total tumor burden [5–7]. These criteria are distinct from RECIST which immediately defines PD at tumor burden increase above the threshold or at the appearance of new lesions. While irRC is increasingly recognized, most trials of immune-checkpoint inhibitors continue to use RECIST1.1 to obtain standardized endpoints used for regulatory approvals in the past decade. Moreover, regulatory authorities have not yet accepted immune-related response evaluations as an endpoint for registrational studies. A need for increased reporting of immune-related responses has been recently acknowledged to address these issues [10], leading to the important initiative by Hodi et al. in their study [1].

## irRC versus RECIST1.1: Methodological differences and their implications

To discuss the results of immune-related responses in their study, the methodological issues of immune-related response evaluations need to be clarified. The original irRC used in the study by Hodi et al. [1] was based on WHO criteria and utilizes bidimensional measurements, quantifying the tumor burden using a product of the longest diameter and the longest perpendicular diameter [2]. On the other hand, RECIST1.1 uses unidimensional measurements, quantifying lesions with the longest diameters except for lymph nodes that use short axis [9, 11]. This methodological difference is a challenge for a direct comparison between irRC and RECIST1.1, because some differences in response evaluations may be due to the difference between unidimensional and bidimensional measures, and may not be due to immune-related response phenomena. Although their motives of comparing irRC against RECIST1.1 are understandable, additional comparisons between irRC and WHO criteria could have contributed to detect the differences purely due to immune-related response phenomena captured by the novel features of irRC, as these two criteria share the same measurement methods with identical thresholds for response and progression.

Measurement variability is another important issue. Multiple prior studies have demonstrated that unidimensional measurements used in RECIST are associated with much less measurement variability compared to bidimensional measurements in WHO/irRC, and therefore can more accurately characterize small tumor burden changes [12–14]. The concept of measurement variability is directly relevant to the threshold values that define response and progression. Notably, the threshold of 25 % bidimensional increase for PD in WHO/irRC can be within the measurement variability, and thus may not necessarily indicate true tumor increase [3, 7, 14]. A prior study by Erasmus et al. reported 43 % misclassification rate for PD using WHO criteria due to interobserver measurement variability [14]. Moreover, smaller lesions are more vulnerable to misclassification, because a small absolute difference in measurements can results in a large percent change [15]. The concept of measurement variability should be carefully applied when interpreting the results of the irRC assessments.

## Pseudoprogression: Definitions, pitfalls, and unmet needs

The study described two types of pseudoprogression; 1) early pseudoprogression with ≥25 % increase at 12 weeks that is not confirmed as PD at the next assessment, and 2) delayed pseudoprogression with ≥25 % increase after 12 weeks that was not confirmed as PD at the next assessment [1]. The observation is interesting because pseudoprogression to date indicated “initial” tumor burden increase followed by subsequent decrease, which mostly falls into “early pseudoprogression”. Although it is intuitive that pseudoprogression does not always occur within 12 weeks of therapy, their observations of delayed pseudoprogression may need to be interpreted with caution in the light of measurement variability. In the spiderplot of 9 delayed pseudoprogressors (Fig. 1B of the JCO article), 7 patients experienced tumor decrease before demonstrating ≥25 % increase from the nadir, including 4 patients who achieved partial response with ≥50 % initial tumor decrease [1]. Subsequent tumor burden increase was relatively small in these patients and right around 25 % comparing to the nadir; this is most notable in a patient whose nadir before pseudoprogression was around –90 % of baseline [1]. It is possible that some cases of “delayed pseudoprogression” are due to measurement variability rather than immune-related response phenomenon. Certain degree of tumor burden fluctuations due to measurement variability are noted during any cancer therapy, particularly when evaluating small tumor burden after initial response using bidimensional measurements that are subject to large variability.

Interestingly, the precise definitions of pseudoprogression have not been actively debated to date. The study defined subsequent tumor reduction as “not confirmed as PD at the next assessment”, and did not require tumor reduction below the partial response threshold. Although this may reflect the concept that “stable disease” is a pattern of response [6], requirement of certain duration of stable disease is likely needed to more rigorously define pseudoprogression. Precise definition will also help to promote the consistent use of the term “pseudoprogression” to describe the unique immune-related phenomenon. Other terms such as “tumor flare” or “disease flare” should be avoided as these terms have been used to describe oncologic conditions that are unrelated to immune-checkpoint inhibitor therapy [16–18].

Another pitfall of the particular version of irRC used in the study is “reset baseline”, which was not used in WHO, RECIST, or in the original irRC; the original irRC mentioned such concept yet defined PD in comparison with nadir [6]. The original irRC commented on a tendency in clinical practice to compare with the most recent prior study when evaluating tumors, as a reason to consider “reset baseline” [6]. However, the serial evaluation of tumor burden dynamics throughout the course of therapy starting at baseline has been the foundation of tumor response criteria over the past 3 decades, and its importance is well-recognized in clinical investigations and practice [19–21]. Indeed, experienced oncologists often review several prior scans to capture the overall tendency of tumor kinetics during therapy. Tumor response criteria offer a unique opportunity to characterize tumor dynamics according to the rigorous methods and standardized language, and baseline burden is a pivotal item [2]. Introducing “reset baseline” without rigorous scientific data supporting the approach has a potential to cause further confusions of immune-related response evaluations and navigate the community away from establishing a consensus. The field currently suffers from a lack of consistencies with the use of different “versions” of immune-related response criteria in clinical trials. A consensus on a unified strategy to effectively evaluate immune-related responses is sorely needed.

Detailed descriptions of pseudoprogression certainly constitute important advances in knowledge provided by the study. There remain unmet clinical needs to be addressed, including predictors and early markers of pseudoprogression that help to differentiate pseudoprogressors and true progressors in earlier course of therapy to facilitate treatment decisions.

## Future directions

Important insights for future directions are provided by Hodi et al., including the use of unidimensional measurement and modifications of RECIST criteria specific to immune-related response evaluations [1]. Such direction is reasonable given the widely accepted use of RECIST in most trials in the past decade. A prior study demonstrated that unidimensional irRC provides highly concordant assessment compared to bidimensional irRC with less measurement variability [3]. Another study reported that modifications of unidimensional irRC according to the revisions in RECIST1.1 regarding the number of target lesions and lymph node assessment also led to concordant immune-related response evaluations [22]. These studies have provided a basis for a direction toward immune-related RECIST1.1 (irRECIST1.1), using unidimensional measurements while maintaining the key features of irRC including new lesion assessments and confirmation for progression. Such approach provides a measure for immune-related response evaluations that allow for “head-to-head” comparisons with the conventional RECIST [3, 7, 22]. The study by Hodi et al. constitutes an important step for further endeavors of immune-related evaluations, where the immune-oncology community needs to bring multidisciplinary expertise together to establish consensus, address unmet needs, and advance the field.

## Ethical approval and consent to participate

Not applicable.

## Consent for publication

Not applicable.

## Availability of supporting data

Not applicable.

## Abbreviations

irRC: immune-related response criteria
JCO: Journal of Clinical Oncology
PD: Progressive disease
PD-1: Programmed-death 1
PR: Partial response
RECIST: Response Evaluation Criteria in Solid Tumors
WHO: World Health Organization
